# Age and Chronicity of Administration Dramatically Influenced the Impact of Low Dose Paraquat Exposure on Behavior and Hypothalamic-Pituitary-Adrenal Activity

**DOI:** 10.3389/fnagi.2017.00222

**Published:** 2017-07-14

**Authors:** Chris A. Rudyk, Jessica McNeill, Natalie Prowse, Zach Dwyer, Kyle Farmer, Darcy Litteljohn, Warren Caldwell, Shawn Hayley

**Affiliations:** Hayley Laboratory, Department of Neuroscience, Carleton University Ottawa, ON, Canada

**Keywords:** paraquat, oxidative stress, aging, Parkinson’s disease, behavior

## Abstract

Little is known of the age-dependent and long-term consequences of low exposure levels of the herbicide and dopaminergic toxicant, paraquat. Thus, we assessed the dose-dependent effects of paraquat using a typical short-term (3 week) exposure procedure, followed by an assessment of the effects of chronic (16 weeks) exposure to a very low dose (1/10th of what previously induced dopaminergic neuronal damage). Short term paraquat treatment dose-dependently induced deficits in locomotion, sucrose preference and Y-maze performance. Chronic low dose paraquat treatment had a very different pattern of effects that were also dependent upon the age of the animal: in direct contrast to the short-term effects, chronic low dose paraquat *increased* sucrose consumption and *reduced* forced swim test (FST) immobility. Yet these effects were age-dependent, only emerging in mice older than 13 months. Likewise, Y-maze spontaneous alternations and home cage activity were dramatically altered as a function of age and paraquat chronicity. In both the short and long-term exposure studies, increased corticosterone and altered hippocampal glucocorticoid receptor (GR) levels were induced by paraquat, but surprisingly these effects were blunted in the older mice. Thus, paraquat clearly acts as a systemic stressor in terms of corticoid signaling and behavioral outcomes, but that paradoxical effects may occur with: (a) repeated exposure at; (b) very low doses; and (c) older age. Collectively, these data raise the possibility that repeated “hits” with low doses of paraquat in combination with aging processes might have promoted compensatory outcomes.

## Introduction

Aging is associated with numerous neurobiological alterations over time, including elevations in hypothalamic-pituitary-adrenal (HPA) axis activity, oxidative radicals, microglia activation and blood-brain barrier (BBB) permeability, coupled with decreases in brain-derived neurotrophic factor (BDNF; Pan, 2011; Jurgens and Johnson, 2012; Derecki et al., 2014; Lucke-Wold et al., 2014). It is well known that these changes can interact to promote “wear and tear” on the individual and modulate susceptibility to environmental insults (Blau et al., 2012; Chapman et al., 2012). Decreased cognitive ability and the emergence of neuropsychological disturbances (e.g., anxiety and depression) often coincide with these age-related biological alterations (Balash et al., 2013; Yochim et al., 2013; Andreescu et al., 2015). Moreover, aging (and the associated neuronal perturbations) is an unequivocal risk factor for Parkinson’s disease, (PD) Alzheimer’s disease (AD) and cerebral stroke (Miller et al., 2009; Vasilevko et al., 2010).

Together with aging, exposure to environmental toxicants likely shapes the evolution of brain pathology. In this regard, paraquat is a commonly used fast acting herbicide that primarily acts as an oxidative stress generator (Jiao et al., 2012; Baltazar et al., 2014), but also has pro-inflammatory and anti-neurogenic consequences (Bobyn et al., 2012; Desplats et al., 2012). Upon entry into the brain, paraquat permeates cortical areas, as well as the hippocampus, olfactory bulbs, and the substantia nigra pars compacta (Peng et al., 2007). Not surprisingly, paraquat can induce neurotoxicity and anti-oxidants may attenuate many such outcomes (McCarthy et al., 2004; Peng et al., 2007; Wang et al., 2011; He et al., 2012). In conjunction with such aberrant biological changes, accumulating evidence suggests that paraquat can also influence neurotransmission in stressor-sensitive brain regions (e.g., prefrontal cortex, hippocampus and locus coeruleus; Chanyachukul et al., 2004; Fernagut et al., 2007; Litteljohn et al., 2008, 2009, 2011; Czerniczyniec et al., 2011; Mitra et al., 2011). We posit that the emergence of paraquat’s deleterious effects might be influenced by the age of the organism upon exposure.

Relatively high doses (10 mg/kg) of paraquat promote the loss of midbrain dopaminergic neurons, reminiscent of what occurs in PD (Liou et al., 2001; Cicchetti et al., 2005; Mangano and Hayley, 2009; Jiao et al., 2012). When considering the upper concentrations of circulating paraquat (~4.8 μg/ml) reported to be associated with survival in cases of human poisoning (Bertsias et al., 2004), together with the LD_50_ for the drug (~4 mg/kg in humans and 130 mg/kg in mice) and the average body size and blood volume (male 76 kg, 5000 ml), the “typical” neurotoxic mouse dose is approaching the range of what might be observed in cases of human poisoning. However, nothing is known regarding the neuronal impact of much lower doses of paraquat and with longer-term exposure. This is important as this likely reflects the more typical situation among pesticide workers and those living in close proximity to such areas.

In the present investigation, we first conducted a relatively short (3 week) paraquat dose-response study to assess HPA functioning (corticosterone, hippocampal glucocorticoid receptor (GR)), along with motor, cognitive and depressive-like behaviors. We next assessed whether long-term exposure (16 weeks) to a relatively low dose of paraquat (1 mg/kg) in “younger” and “older” aged mice (beginning at 5 or 13 months of age, respectively) differentially affects biological and behavioral stressor consequences. We additionally sought to determine whether this paraquat regimen influenced expression of the trophic factor, BDNF or the anti-oxidant related factor [Nuclear factor erythroid 2-related factor 2 (Nrf2)]. It was initially hypothesized that the short-term effects of paraquat would be augmented by the long term exposure regimen. Instead, we observed a highly complex profile of behavioral and corticoid changes that suggest adaptive alterations occurred with repeated low-dose exposure to the toxicant in aged animals.

## Materials and Methods

### Paraquat Dose Response

#### Animals and General Experimental Design

Briefly, 36 male C57/BL6 mice (Charles River, Laprarie, QC, Canada) aged 3 months were single housed in standard (27 cm × 21 cm × 14 cm) fully transparent polypropylene cages and were acclimated to the Carleton University vivarium for 2 weeks. Mice were maintained on a 12-h light/dark cycle in a temperature-controlled (21 degrees) environment with *ad libitum* food and water. All experimental test protocols were approved by the Carleton University Committee for Animal Care and in very strict accordance with the guidelines outlined by the Canadian Council for the Use and Care of Animals in Research. All work was approved by these ethics boards. All behaviors were performed between the hours of 8:00 am and 4:00 pm.

#### Injection Protocol

**Figure 1:**
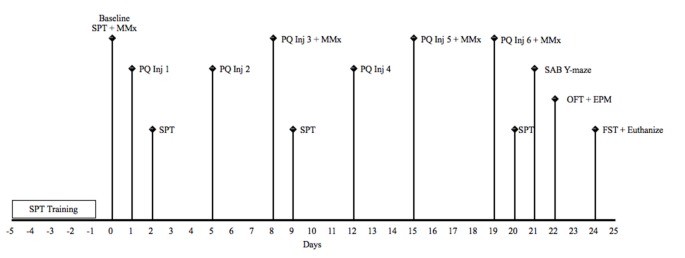
Schematic timeline for study 1. EPM, elevated plus maze; FST, forced Swim test; Inj, injection; MMx, micromax; OFT, open field test; PQ, paraquat (1 or 10 mg/kg); SAB, spontaneous alternation behavior; SPT, sucrose preference test.

Animals received intraperitoneal (i.p) treatment of paraquat [1,1′-dimethyl-4,4′-bipyridinium dichloride; Sigma Aldrich, 1 mg/kg (low dose) or 10 mg/kg (high dose)] or an equivalent volume of physiological saline (Sigma; 12/group), two times per week for three consecutive weeks. All injections occurred in the morning beginning at 8:30 am. Mice were sacrificed 5 days following the last paraquat or saline injection via rapid decapitation 5 min following the last behavioral test. A timeline for the study is depicted in Figure 1.

#### Home Cage Locomotor Activity

Spontaneous home cage locomotor activity was measured over a complete 12 h light/dark cycle using our Micromax (MMx) infrared beam-break apparatus (Accuscan Instruments, Columbus, OH, USA), as previously described (Litteljohn et al., 2014). Total home cage locomotor activity is determined based on the number of infrared beam-breaks an animal makes based on 16 infrared wavelengths originating external to the animal’s home cage. Briefly, following a 30 min acclimation period in our behavioral testing room post nestlet removal, measurements of 12-h home cage motor activity occurred once at baseline, then again the evening of the 3rd, 5th and 6th injection.

#### Sucrose Preference Test

In order to assess whether paraquat exposure at different doses is associated with depressive-like non-motor behavior (very common in PD patients), a sucrose preference test (SPT) was conducted as a measure of anhedonia (Willner et al., 1987). All animals received baseline sucrose preference training for a period of 5 days prior to the first injection and the SPT was given 1 day following the 1st, 3rd and 6th injection. Briefly, on baseline and testing days, animals were simultaneously exposed to two 200 ml bottles containing sucrose solution or tap water randomly placed approximately 5 cm apart from each other. Animals received 5 days of baseline testing in which they received 2 days of 2% sucrose solution followed by 3 days of a 1% sucrose solution post-acclimation. The amount of solution consumed was determined based on bottle weights before and 24 h after placement. All solutions were made fresh each day. Preference for the sucrose solution was calculated according to the following formula: sucrose intake/(sucrose intake + water intake) * 100.

#### Spontaneous Alternation Behavior Y-maze

In order to assess working memory and executive function impairment induced by low or high dose paraquat exposure, an adapted version of the Y-maze was used, as outlined by Wall and Messier (2001). The Y-maze consisted of three arms each 40 cm long × 3 cm wide enclosed by 13 cm high walls which converged on an equilateral triangle at the center. Animals were tested in this version of the Y-maze 2 days following the final injection. Briefly, each animal was individually placed at random in one of three enclosed arms for a total of 8 min. Alternate arm returns (AAR), same arm returns (SAR), and spontaneous alternation behavior (SAB) performance was recorded when an animal had placed all four paws in the arm runway outside of the center triangle. SARs were defined as when an animal left a previously entered arm and then returned to the same arm, with at least two paw entry into the center triangle and without total entry into another arm. AAR was defined as when an animal returned to a previously entered arm after four paw entry into another arm (for example arm A to B and then back to arm A). SAB performance was defined when an animal had entered each arm with four paws in a sequential order without returning to a previous arm (for example arm A to B followed by arm B to C). For appropriate data analysis, scores were expressed as percentages, in order to not bias any results affected by the total number of arm entries. Thus the following equations were used: %SAR = total number of same arm returns/total number of arm entries * 100%, AAR = alternate arm returns/total arm entries * 100, and %SAB = total number of sequential alternations/total arm entries * 100.

#### Open Field Test

The open field test (OFT) was used to assess the influence of paraquat dose on anxiety-like behavior, as previously described by Salmaso et al. (2016). The OFT was conducted 3 days following the last paraquat/saline injection. Briefly, an open white Plexiglas arena (26 × 48 cm) was used, and divided into pre-defined zones (outer and inner/center). Each animal was placed at random in one of four corners and behavior was recorded for a period of 20 min. The amount of time spent, distance traveled, speed, and freezing behavior in each zone was recorded using our automated video-tracking system (Any-Maze software, version 4.71). Anxiety-like behaviors measured included the amount of time an animal spent in either of the pre-defined zones, the amount of time spent immobile in the zones and the frequencies of entry into each zone. The arena was cleaned with 10% EtOH between trials.

#### Elevated Plus Maze

To further measure anxiety-like behavior, the elevated plus maze (EPM) was used, as previously described (Salmaso et al., 2016). The EPM consists of four arms (24.8 cm long × 7 cm wide) with two closed arms enclosed by 21 cm high walls and elevated approximately 60 cm off the surface of the floor. Three days following the last injection and 1 h following the OFT, each animal was individually placed in the center of the four-arm maze, and behavior was recorded for a total of 6 min using Any-Maze software, version 4.71. Anxiety-like behaviors measured included the percent time spent in the open vs. closed arms during the first 5 min of the test.

#### Forced Swim Test

In order to further assess depressive-like effects (i.e., behavioral despair) that may be induced by differential doses paraquat exposure, a modified version of the Porsolt et al. (1977) forced swim test was used (FST; as previously described by Litteljohn et al., 2010). Mice were individually placed in a glass cylinder 20 cm in diameter that contained temperature controlled water (25 ± 1°C) at a depth of approximately 20 cm for a total of 6 min. Time immobile during the last 4 min of the test was recorded on our camera and scored by an independent observer blind to all experimental conditions. Immediately following the task, animals were dried off, placed in their home cage, and quickly transferred to necropsy where rapid decapitation was performed.

#### Brain Extraction

Five minutes following the final behavioral task (between 8:30 am and 11:00 am), mice were dried and tissue was collected for western blot analysis. For rapidly decapitated animals, a chilled microdissecting block that contained slots (0.5 mm apart) for single edged razor blades was used. Brains were quickly excised and the hippocampal region was micro-punched from coronal brain sections in order to assess the effects of differential doses of paraquat exposure on glucocorticoid and phosphor-GR expression. The tissue was immediately frozen upon dissection and stored at −80°C until processing.

##### Western blot

Tissue punches collected from the hippocampus were used to detect levels of brain the GR (~86 kDa; Santa Cruz) and the phospho-glucocorticoid receptor (pGR (Ser211), ~95 kDa; a marker of activated GR; Cell Signalling Technology). Briefly hippocampal whole cell lysates were homogenized in Radioimmunoprecipitation Assay (RIPA) buffer [50 mM Tris (pH 8.0), 150 mM sodium chloride, 0.1% sodium dodecyl sulfate (SDS), 0.5% sodium deoxycholate and 1% Triton X-100] mixed with 1 tablet of Complete Mini EDTA-free protease inhibitor (Roche Diagnostics, Laval, QC, USA, Cat #11 836 170 001) per 10 mL of buffer and then sonicated for 30 s in ice cold water. The lysed cells were then centrifuged at 5000 RPM with a table top microcentrifuge for 10 min at 4°C. The supernatant was then extracted and protein concentration was determined using bicinchoninic acid (BCA) method (Thermo Scientific, Cat #23227). Following protein concentration determination supernatant was placed in 5× loading buffer (containing; 5% glycerol, 5% β-mercaptoethanol, 3% SDS and 0.05% bromophenol blue) and the protein was denatured when placed in a 5 min heating block at a temperature of 105°C. Following this step, samples were placed in a −20°C freezer until processing commenced.

On the first day of analysis, proteins were separated using SDS-polyacrylamide gel electrophoresis (SDS-PAGE). Briefly the SDS-PAGE gel (7.5%) containing the separating buffer (370 mM Tris-base (pH 8.8), 3.5 mM SDS), and the stacking buffer (124 mM Tris-base (pH 6.8), 3.5 mM SDS), were placed in running buffer (25 mM Tris-base, 190 mM glycine, 3.5 mM SDS) and samples, along with the Precision Plus Protein™ Standards Dual Color (Bio-Rad, Hercules, CA, USA, Cat #161-0374), were loaded into the Arcylamide gel (7.5%) for molecular weight determination at 140 volts. After electrophoresis, proteins were transferred for 1 h at 4°C at 100 volts in transfer buffer solution (25 mM Tris-base, 192 mM Glycine, 20% methanol), onto PVDF (Bio-Rad, Cat #162-0177). Thereafter, membranes were dried overnight.

In order to determine total protein, after a brief methanol rinse (5 s), membranes were incubated in a reversible stain, REVERT^®^ (Li-COR Biotechnology) for a period of 5 min followed by placement into a wash solution (6.7% Glacial Acetic Acid, 30% Methanol, in water) two times 2 min each. Membranes were then quickly rinsed with distilled water and imaged on a LI-COR Odyssey imaging system on the 700 channel for an exposure period of 2 min. Membranes were rinsed immediately post imaging in tris buffered saline (TBS; pH 7.5 (2 × 5 min)) buffer and then placed in blocking solution (0.5% fish gelatin (Sigma) in TBS) for 90 min. After blocking, membranes were incubated in either a rabbit anti-GR primary antibody (1:500) or rabbit anti-pGR (1:1000), as well as mouse anti-actin (ThermoFisher) for a period of 60 min in 0.05% fish gelatin in TBS with 0.1% tween. Any unbound antibody was removed using 15 mL of TBS-T/membrane at room temperature four times 5 min each. Membranes were then incubated for 1 h in infrared conjugate directed against the species the primary antibody was raised in (rabbit 800, LI-COR) at a concentration of 1:20,000 in 0.5% fish gelatin solution containing 0.2% tween and 0.01%SDS. Membranes were then washed in TBS-T (4 × 5 min) followed by 2 × 5 min washes in TBS and read on the LI-COR Odyssey system at 800 nm for 6 min.

##### Corticosterone (CORT) assay

Differences in CORT levels were obtained with an ELISA CORT determination assay (Corticosterone #900-097, Lot# D1260724) using trunk blood collected immediately after decapitation. All blood samples were collected in EDTA-coated Eppendorf tubes, after which they were spun for 20 min at 4°C (20,000 *g*). After serum collection samples were immediately frozen and stored at −80°C. CORT determination was then performed using our microplate reader and quantified.

### Aging and Chronic Low-Dose Paraquat Exposure

#### Animals

**Figure 2:**
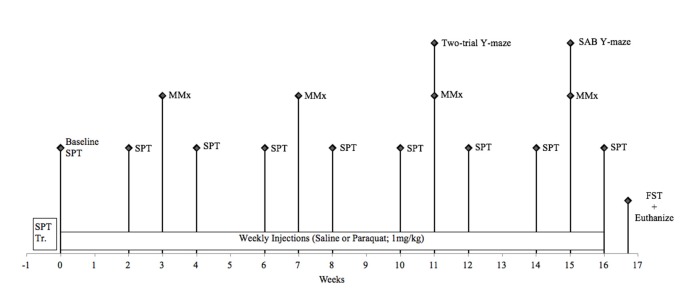
Schematic timeline for study 2. FST, forced swim test; MMx, micromax; SAB, spontaneous alternation behavior; SPT, sucrose preference test.

Forty-two male C57/BL6J mice (Jackson Laboratories, Bar Harbor, ME, USA) aged 5 months (operationally defined as “younger” cohort) and 13 months (operationally defined as (“older” cohort) were housed in standard (27 cm × 21 cm × 14 cm) fully transparent polypropylene cages. The entire study lasted 4 months; thus, mice were either 9 months (“younger” cohort; 5–9 months) or 17 months (“older” cohort; 13–17 months) of age at the time of sacrifice. All mice were initially obtained at 6–8 weeks of age and singly housed in the same conditions as Study 1. All animal procedures were performed in accordance with Carleton University animal care committee’s regulations. A timeline for this study can be seen in Figure 2.

#### Treatments and General Procedures

Animals received i.p. injections of paraquat (1 mg/kg) or an equivalent volume of physiological saline once per week for 16 consecutive weeks (*n* = 10–12/group). This dose was based on previous studies demonstrating no peripheral toxic effects and is believed to be below the concentration required to produce degeneration of dopamine neurons (McCormack et al., 2002; Yin et al., 2011). Indeed, our primary goal was to evaluate the consequences of chronic low dose oxidative stress exposure in the context of advancing age and not to model the frank neurodegenerative effects seen in previous PD models.

### Behavioral Tests

#### Home Cage Locomotor Activity

Spontaneous locomotor activity was measured over a complete 24 h light/dark cycle using our Micromax (MMx) infrared beam-break apparatus (Accuscan Instruments, Columbus, OH, USA), as described above. Briefly, following a 30-min acclimation period in our behavioral testing room, measurements of locomotor activity occurred 1 h after paraquat or saline exposure one time per month during the 16-week paradigm (i.e., weeks 3, 7, 11 and 15).

#### Two-Trial Y-maze

In order to test spatial memory retention performance, the discreet two-trial Y-maze was used (Dellu et al., 2000). During this task, animals were first individually placed into one of two arms in the Y-maze for a period of 5 min, with the third arm blocked by a divider at random. Following a 30-min interval, the divider was removed and animals were allowed to freely explore all three arms of the maze for another 5 min. In order to analyze the task, the time spent exploring the novel vs. familiar arms was calculated as the ratio (time spent in the novel arm/(time spent in the novel + adjacent arms)) × 100. Time spent in novel arms is believed to be a measure of spatial memory and retention and was expressed in percentages in order to not bias any locomotor results (calculated as the number of arm entries during the acquisition trial). This task was held in our behavior room on week 11 of the behavioral paradigm immediately after locomotor measurements were taken and 1 day after paraquat or saline injection. All arms were cleaned with 10% ethanol between trials.

#### Spontaneous Alternation Behavior Y-maze

In order to test for working memory performance, the SAB Y-maze was used as described in Study 1. This version of the Y-maze was performed on Week 15 immediately after locomotor testing. All arms were cleaned with 10% ethanol between trials. All trials were recorded on our camera and later scored by an observer who was naïve to the study.

#### Sucrose Preference Test

In order to assess whether or not low-dose chronic paraquat exposure is associated with depressive-like behaviors, a SPT was administered as a measure of anhedonia, as described in study 1. Briefly, every other week, 1 day following paraquat or saline injections mice were simultaneously exposed to two 200 ml bottles containing 1% sucrose solution or tap water randomly placed approximately 5 cm apart from each other. All solutions were made fresh each day and bottle weight measurements were taken before and 12 h after overnight testing in order to determine sucrose preference.

#### Forced Swim Test

In order to assess depressive-like effects (i.e., behavioral despair) that may be induced by chronic paraquat exposure, a modified version of the Porsolt et al. (1977) FST was used as described in Study 1. Immediately following the task, animals were dried off and placed in their home cage and quickly transferred to necropsy where rapid decapitation was performed.

### Biological Outcome Measures

#### Brain Dissection and Plasma Collection

All mice were sacrificed 5 days following the last paraquat or saline injection. Five minutes after the final behavioral task (between 9:00 am and 11:00 am), mice were rapidly decapitated and brains and trunk blood were collected (as described in Study 1) in order to evaluate plasma corticosterone and hippocampal protein expression (i.e., phosphorylated glucocorticoid receptors (pGR), BDNF and Nrf2 via radioimmunoassay and Western blot analyses, respectively). Tissue was immediately frozen and stored at −80°C until processing. Trunk blood was centrifuged at 3600 rpm (8 min) in order to collect plasma and was stored at −80°C until corticosterone determination.

#### Corticosterone Determination

In order to determine concentration levels of corticosterone in plasma, a commercial radioimmunoassay kit (ICN Biomedicals Inc., USA) was used. Assays were performed in a single run to prevent inter-assay variability; intra-assay variability was <10%.

#### Western Blots

Tissue samples were collected from the hippocampus to detect levels of mature BDNF (~14 kDa), Nrf2 (~68 kDa) and pGR (~95 kDa) as previously described (Griesbach et al., 2012). Primary antibodies for BDNF (1:300, Abcam), Nrf2 (1:500, Abcam) and pGR (1:500, Cell Signaling Technology) were used and β-actin (~42 kDa; 1:10,000, Sigma) was applied as a loading control. Protein determination and Western blot procedures for hippocampus whole cell lysates were carried in a procedure identical to study 1 (i.e., from tissue homogenization—protein PVDF membrane transfer).

Following transfer, membranes were blocked for 1 h with gentle shaking in a solution of non-fat dry milk (5% w/v) dissolved in TBS-T buffer (10 mM Tris-base (pH 8.0), 150 mM sodium chloride, 0.5% Tween-20). The membranes were then incubated with a rabbit anti-BDNF, rabbit anti-Nrf2 and rabbit anti-phospho GR primary antibody diluted in blocking solution at room temperature for 1 h. Any unbound antibody was removed using three 10 min washes of 15 mL TBS-T at room temperature. Membranes were incubated on a shaker for 1 h at room temperature with HRP (horseradish peroxidase) conjugated anti-rabbit (1:5000) secondary antibody and washed again with TBS-T. BDNF, Nrf2 and pGR were then visualized using Western Lightning Plus chemiluminescent substrate (Perkin Elmer, Waltham, MA, USA, Cat #.NEL102001EA) and by exposure to Kodak X-OMAT film. The molecular weights of proteins were estimated using Precision Plus Protein Kaleidoscope Standards (Bio-Rad, Hercules, CA, USA). Protein bands were quantified by densitometry using AlphaEase software (AlphaEase FC V.3.1.3., Alpha Innotech Co., San Leandro, CA, USA) and normalized to actin.

#### Data Analysis

For Study 1, all data was analyzed using a one-way analysis of variance (ANOVA) for paraquat dose treatment comparisons, with significant interactions further analyzed by means of Fisher’s planned comparisons (*p* < 0.05). For Study 2, all data was analyzed using a 2 (paraquat vs. saline treatment) by 2 (younger vs. older cohorts) two-way ANOVA design followed by Fisher’s planned comparisons (*p* < 0.05) where appropriate. Additionally, for both studies, the analyses of SPT and total home cage locomotor activity, was completed using appropriate repeated measures ANOVA’s conducted with *Time* as the 3rd independent variable followed by *post hoc* analysis. Data is presented in the form of mean ± standard error mean (mean ± SEM). All data was analyzed using the statistical software StatView (version 6.0) and differences were considered statistically significant when *p* < 0.05.

## Results

### Study 1: Paraquat Dose-Response

#### Spontaneous Home Cage Locomotor Activity

**Figure 3:**
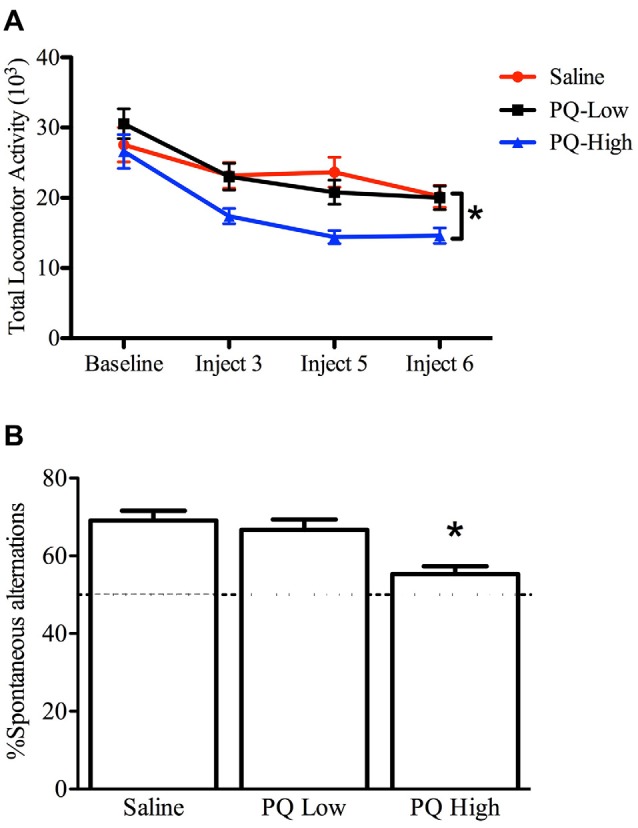
Dose-dependent effects of short term paraquat treatment on home cage activity and Y-maze performance. As depicted in **(A)**, the higher dose of paraquat (PQ-High; 10 mg/kg; blue line) significantly reduced home cage activity, relative to mice treated with the low toxicant dose (PQ-Low; 1 mg/kg; black line) or saline (red line). As depicted in **(B)**, the high paraquat dose likewise reduced the number of spontaneous alternations in a Y-maze task, suggesting some degree of cognitive deficit. *p < 0.05, relative to saline or low-dose paraquat treated mice.

The one-way repeated measures ANOVA conducted to compare the effect of paraquat dose on total home cage activity after three, five and six injections demonstrated that paraquat reduced home cage activity levels (*F*_(2,33)_ = 4.631, *n* = 12/group, *p* < 0.05). The follow-up comparisons clearly revealed that the locomotor reduction in the beam-breaks was confined to mice that received the high dose of paraquat (*p* < 0.05; Figure 3).

#### Working Memory in a Spontaneous Alternation Y-maze

A significant main effect was evident for paraquat treatment with regard to percent spontaneous alternations (*F*_(2,33)_ = 9.53, *n =* 12/group, *p* < 0.001). Indeed, paraquat did appear to dose-dependently disrupt working memory, as indicated by the reduction of alternations provoked by the high paraquat dose group (*p* < 0.05; Figure 3).

#### Anxiety-Like Behavior in the Open Field and Elevated Plus Maze Tests

No dose-dependent effects of paraquat treatment were evident on anxiety-like behavior measures using the elevated plus maze or the OFT.

#### Depressive-Like Behaviors in Sucrose Preference and Forced Swim Tests

All animals included showed an at least 70% preference for sucrose over water by the end of the baseline period. The results of the repeated measures ANOVA analyses indicated that there was no significant effect of paraquat at either of the doses administered.

**Figure 4:**
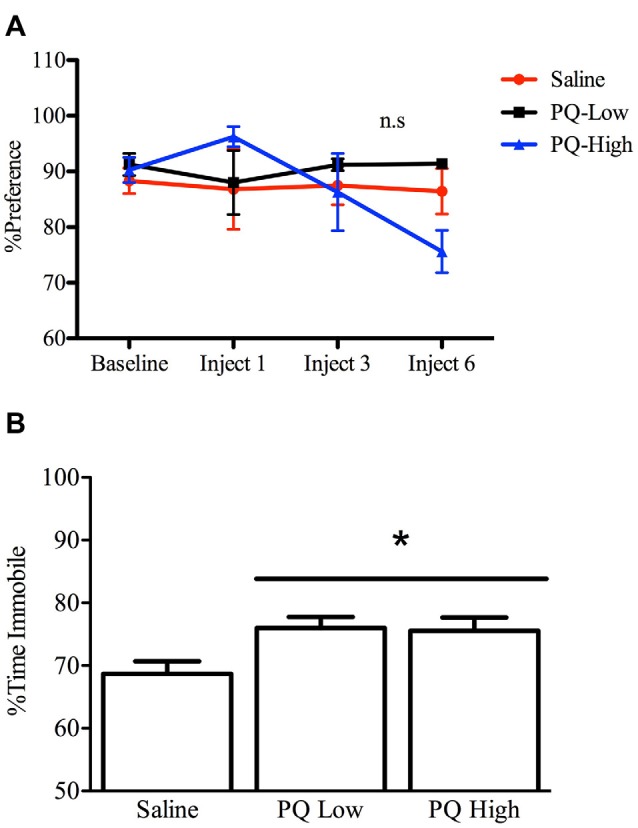
Dose-dependent effects of short term paraquat exposure on force swim immobility but not sucrose preference. As shown in **(A)**, short term low dose (PQ-Low; 1 mg/kg) or high dose (PQ-High; 10 mg/kg) paraquat exposure did not influence sucrose preference at any time point (n.s = not significant). As shown in **(B)**, both the 1 and 10 mg/kg doses of paraquat effectively increased the time immobile in a forced swim test. *p < 0.05, relative to saline treated mice.

Paraquat however, significantly affected FST performance *F*_(2,32)_ = 4.25, *n* = 12/group, *p* < 0.05). Interestingly, both the low and high doses of paraquat significantly increased time immobile, relative to controls (*p* < 0.05; Figure 4). Thus, paraquat appears to differentially affect tests that tap into anhedonic (sucrose preference) vs. “learned helplessness” (forced swim).

#### Plasma Corticosterone

**Figure 5:**
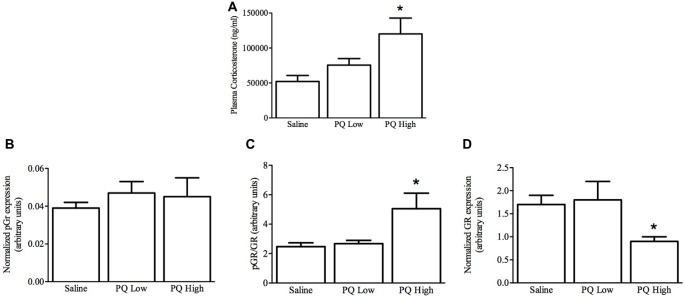
Dose-dependent effects of short term paraquat administration on plasma corticosterone and hippocampal glucocorticoid receptor (GR) levels. As shown in **(A)**, the higher paraquat dose (PQ High; 10 mg/kg) relative to the lower dose (PQ Low; 1 mg/kg) significantly increased plasma corticosterone levels. At the same time, paraquat did not increase levels of the active form of GR (pGR; **B)**, but the higher toxicant dose did increase levels of pGR relative to GR **(C)**. However, given that the higher paraquat dose decreased levels of total GR **(D)** within the hippocampus, pGR levels were unaltered by the toxicant. *p < 0.05, relative to saline and low dose paraquat treated mice.

As shown in Figure 5, paraquat treatment influenced plasma corticosterone levels (*F*_(2,15)_ = 5.32, *n* = 9/group, *p* < 0.01). The follow-up comparisons indicated that again paraquat dose-dependently increased plasma corticosterone concentrations, such that only the high dose was effective in this regard (*p* < 0.05).

#### Hippocampal GR Levels

Paraquat exposure significantly influenced hippocampal levels of GR (*F*_(2,15)_ = 4.51, *n* = 9/group, *p* < 0.05) but not pGR *F*_(2,15)_ = 0.263, *p* > 0.05). Specifically, as shown in Figure 5, and confirmed by follow-up comparisons, the high dose of paraquat reduced hippocampal GR levels, relative to saline or low dose paraquat treatment (*p* < 0.05). Similarly, the ratio of pGR to GR was significantly affected by paraquat *F*_(2,15)_ = 4.92, *p* > 0.05, and this effect was restricted to an increase provoked by the high dose (*p* < 0.05). Yet, it was clear that overall GR reduction was driving this effect.

### Study 2: Age-Dependent Effects of Chronic Low-Dose Paraquat Exposure

#### Spontaneous Home Cage Locomotor Activity

**Figure 6:**
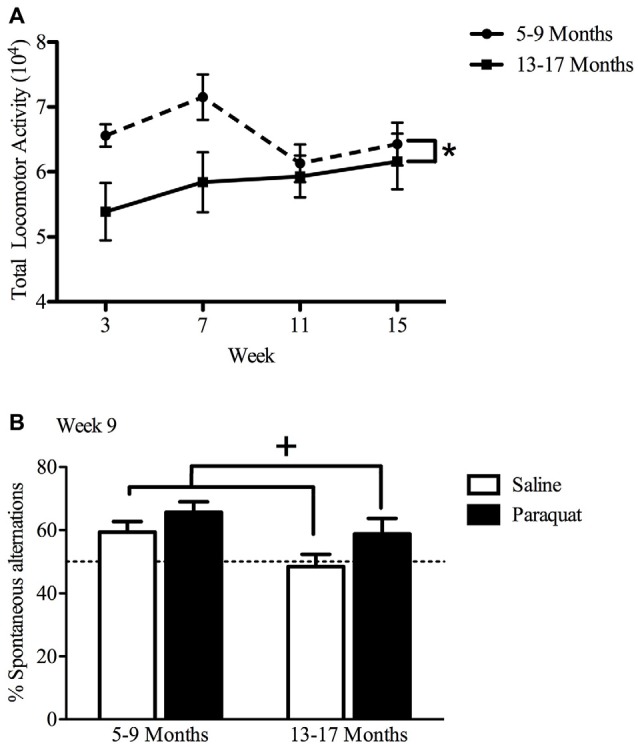
Effect of long-term (low dose) paraquat exposure and age on locomotor activity and spontaneous alternation version of a Y-maze. Irrespective of paraquat treatment, the older cohort of mice (13–17 months) showed reduced total home-cage locomotor activity **(A)** in comparison to the younger cohort (5–9 months). *p < 0.05 relative to the younger cohort (5–9 months; data presented collapsed over paraquat treatment for better representation). In the absence of a significant age effect, paraquat treatment increased the number of spontaneous alternations in a Y-maze **(B)**. Yet, although not significant, the magnitude of this effect appears greater in the aged mice. Dotted line indicates chance performance. ^+^*p* < 0.05 relative to saline treated mice (collapsed over age).

Overall, results of the repeated measures two-way ANOVA demonstrated only age-related differences in home cage locomotor activity, with older mice (age 13–17 months) displaying lower home cage activity levels (*F*_(1,32)_ = 4.81, *n* = 9–11/group, *p* < 0.05), compared to the younger cohort (age 5–9 months; Figure 6). Exposure to paraquat treatment did not significantly influence home cage locomotor activity in either group.

#### Working Memory in a Spontaneous Alternation Y-maze

The paraquat treatment group showed a significant main effect in the percentage of spontaneous alternations (*F*_(1,32)_ = 5.29, *n* = 7–10/group, *p* < 0.05), with paraquat-treated animals making more spontaneous alternations. Although no significant main effect of Age or Age by Paraquat treatment interaction was observed, it was clear that a reduction in the number of spontaneous alternations was most apparent in the saline treated mice who began the injection regimen at 13 months (Figure 6). From another perspective, as shown in Figure 4, paraquat appeared to enhance spontaneous alternations in the older aged animals. In all cases, except the saline treated older mice, performance was statistically above chance (50%).

#### Spatial Memory in a Two-Trial Y-maze

No significant main effects, or interaction with regard to Age or Paraquat treatment was observed on % time spent in novel arm in the two-trial Y-maze cognitive task.

#### Anhedonia in a Sucrose Preference Test

**Figure 7:**
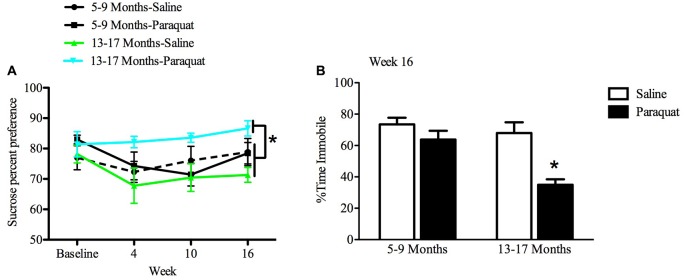
Effect of long-term (low dose) paraquat treatment and age on sucrose preference and forced swim performance. As depicted in **(A)**, paraquat treatment augmented sucrose preference in the older cohort (13–17 months) relative to the remaining three groups. In contrast, the younger cohort of mice (5–9 months) that received paraquat (black line) did not differ from their saline treated counterparts (dotted black line) or saline treated aged animals (green line). Time spent immobile in the FST is shown in **(B)**. The older cohort of mice exposed to the low dose, chronic paraquat regimen (right black bar) spent significantly less time immobile than the other three groups. *p < 0.05 relative to all other groups.

All animals showed an at least 70% preference for sucrose over water by the end of the baseline period. While a relatively long interval elapsed between sucrose preference testing sessions, preference was nevertheless maintained across all groups 2 weeks after baseline measurements. The results of the repeated measures ANOVA analyses indicated that there was a significant Age by Paraquat interaction in sucrose preference scores (*F*_(1,28)_ = 8.54, *n* = 7–9/group, *p* < 0.01). Specifically, as shown in Figure 7, older mice exposed to paraquat beginning at 13 months had greater sucrose preference than their age-matched counterparts administered saline (*p* < 0.05). No such difference in sucrose preference was observed among the middle-aged animals as a function of paraquat exposure (Figure 7).

#### Forced Swim Test Performance

A two-way ANOVA revealed a significant Age by Paraquat treatment interaction on the percent of time animals spent immobile (*F*_(1,32)_ = 4.16, *n* = 7–11/group, *p* < 0.01). As shown in Figure 7 and confirmed by Fisher’s planned comparisons, mice from the older cohort (13–17 months) administered paraquat displayed a significant reduction in time immobile in comparison with the saline-treated younger cohort (5–9 months; *p* < 0.01) and same age counterparts (*p* < 0.05), as well as in relation to younger mice exposed to paraquat (*p* < 0.05).

#### Plasma Corticosterone

**Figure 8:**
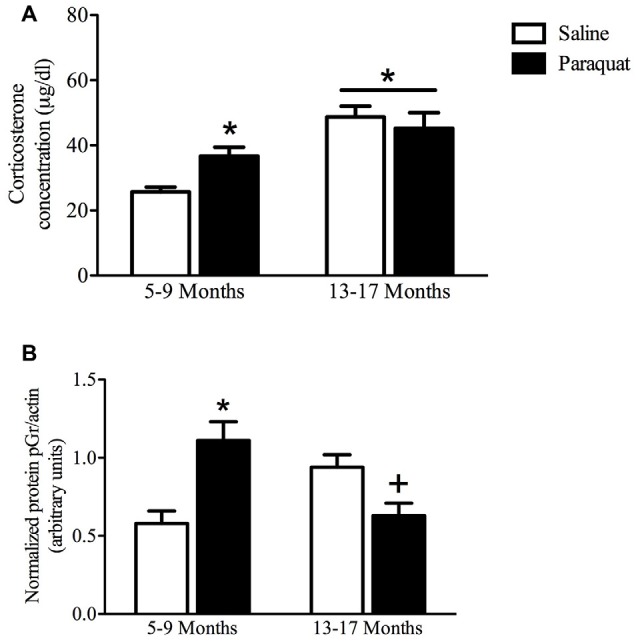
Plasma corticosterone and hippocampal pGR protein as a function of age and long-term (low dose) paraquat treatment. As shown in **(A)**, the older cohort (13–17 months) displayed elevated corticosterone levels (collapsed over paraquat treatment). Paraquat treatment elevated corticoid levels; but interestingly, this effect was restricted to the younger cohort (5–9 months). *p < 0.05 relative to the saline treated younger cohort. As displayed in **(B)**, paraquat robustly increased hippocampal protein levels of pGR in the younger cohort in comparison to age matched saline treated mice, however, it significantly reduced levels in mice from the older cohort, relative to their saline injected counterparts. *p < 0.05 relative to saline treated mice from the younger cohort, ^+^*p* < 0.05 relative to saline treated mice form the older cohort.

As shown in Figure 8, a significant Age by Paraquat treatment interaction was evident with respect to plasma corticosterone levels (*F*_(1,33)_ = 5.69, *n* = 7–11/group, *p* < 0.05). The follow-up comparisons indicated that corticosterone concentrations were significantly increased by paraquat treatment in the younger mice (*p* < 0.05), whereas no such effects were apparent in the older cohort. However, age itself had a dramatic effect on corticosterone with aged animals overall showing a marked elevation of the hormone that exceeded that of the younger mice (*p* < 0.01).

#### Hippocampal pGR Levels

Western blot analysis of hippocampal pGR protein levels revealed a significant Age by Paraquat treatment interaction (*F*_(1,26)_ = 20.19, *n* = 6–9/group, *p* < 0.01; Figure 8). Follow-up with Fisher’s planned comparisons revealed that mice exposed to the low-dose paraquat treatment in the younger cohort had significantly higher hippocampal pGR protein levels than their saline exposed counterparts (*p* < 0.01). Moreover, as shown in Figure 8, age itself was associated with a rise in pGR expression, yet paraquat treatment in the older cohort actually reduced this elevation (*p* < 0.05).

Additional Western blot analyses of BDNF and Nrf2 levels within the hippocampus yielded no significant differences between any of the treatment groups. However, we should mention that our Nrf2 antibody was designed to detect the expected molecular weight of 68 kDa; yet it was recently reported that the protein yielded a band with a molecular weight of 95–110 kDa (Lau et al., 2013). That said, using our procedures, we reliably detected a 68 kDa band with negligible higher weight signals.

## Discussion

Given that paraquat is essentially an oxidative stress generator, it is not surprising that extensive brain changes in oxidative and inflammatory signaling factors have also been reported, along with a variety of behavioral disturbances (Litteljohn et al., 2009; Mangano et al., 2011). However, these studies were conducted over a relatively short period of time in young animals and involved high doses of paraquat that can kill dopaminergic neurons (McCormack et al., 2006; Mangano et al., 2011). In contrast, the present investigation assessed the effects of a substantially lower paraquat dose (1 mg/kg; 1/10th of what we use in our Parkinsonian animal model studies; e.g., Mangano and Hayley, 2009). Moreover, we used older mice (either 5 or 13 months of age at the beginning of the present study, compared to 2–3 month-old animals in previous studies) and longer-term exposure (4 months compared to previous studies involving 3 weeks of paraquat treatment, e.g., Mangano and Hayley, 2009; Mangano et al., 2011). We were particularly interested in whether any behavioral and biological (HPA functioning) indices of stressor reactivity effects would be augmented over time in the older animals (given that aging itself is associated with cumulative oxidative and inflammatory effects; Vida et al., 2014).

While short-term (3 weeks) paraquat treatment had dose-dependent behavioral and hormonal effects similar to that of traditional stressors, long-term (4 months) treatment with low doses of paraquat (that themselves have few short-term effects) provoked a complex pattern of behavioral and hormonal changes that were quite unexpected. In fact, the chronic low-dose paraquat treatment didn’t affect home cage activity, but had effects in the Y-maze, forced swim and SPTs that were the opposite of what one might predict, based on the known deleterious effects of the toxicant. These animals also displayed marked changes in HPA functioning, suggesting that a general stress response was induced by both paraquat and aging. These findings raise the possibility that adaptive responses were engendered by paraquat, or essentially that the repeated low levels of oxidative stress provoked by the toxicant might have triggered adaptive cellular stress pathways.

It is well known that certain toxicants can have U-shaped or inverted U-shaped dose-response relationships that follow the concept of hormesis, wherein higher doses have toxic effects but lower concentrations can actually impart beneficial actions (Wang, 2012). For instance, it was previously reported that low concentrations of paraquat could promote an adaptive stress response to increase survival of knockdown parkin flies (a genetic insect model of Parkinsonism; Mattson, 2008; Bonilla-Ramirez et al., 2013). Yet, no other such reports exist in other model organisms, and it is entirely unknown as to whether hormetic effects might be observed at the behavioral or systems level with repeated *in vivo* injection of the herbicide in rodents. It is of interest that repeated mild stress challenges have been reported to induce anti-aging effects that were posited to stem from changes in proteasomal and anti-oxidant functions (Rattan et al., 2004; Mattson, 2014). Importantly, however, our attempt to link possibly “adaptive” trophic (BDNF) and anti-oxidant (Nrf2) hippocampal factors to the observed paraquat and aging responses was unsuccessful, as these factors were unaffected by any of the treatments (data not shown).

While the high dose of paraquat reduced hippocampal GR, neither the high nor low doses affected its phosphorylated active form, pGR, in the short term study. In contrast, the long-term low dose paraquat treatment increased hippocampal pGR in the “younger” 5–9 month old mice of the chronic study. Most interesting however, the long-term low-dose paraquat treatment reduced hippocampal pGR in the “older” 13–17 month mice. Conceivably, the herbicide might influence negative feedback aspects of HPA functioning in mice that received the “added hit” of pesticide exposure in the context of older age. Indeed, plasma corticosterone levels and hippocampal pGR expression were increased by both paraquat and aging. However, most intriguingly, the combination of older age and paraquat reduced hippocampal pGR expression back to basal levels.

It is possible that such GR changes could reflect compensatory or adaptive processes that were engaged (possibily in response to chronically elevated corticosterone levels). This raises the possibility that with age the normal HPA genetic regulatory apparatus might be altered which in turn, could either facilitate or hinder adaptive homeostatic processes. Such processes could essentially moderate corticosterone secretion in an attempt to try and restore homeostasis. It is also possible that while the combined paraquat + aging variables did not further increase the overall magnitude of the hormonal response (possibly resulting from a ceiling effect, given that the levels were exceedingly high; approaching 60 ug/dl), the kinetics of the response might have been altered. Yet, we cannot definitively speak to the time-course for variations in the hormone given the single sampling time.

The present findings are in agreement with our proposition that paraquat acts as a systemic stressor, much the same as pro-inflammatory cytokines (Rudyk et al., 2015), and that its HPA effects could contribute to depressive or other co-morbid features that are often evident in neurodegenerative diseases, such as PD. Indeed, the observed paraquat-induced dose-dependent corticosterone elevation, coupled with the decrease in total hippocampal GR is consistent with what has been observed with psychological stressor exposure (Füchsl and Reber, 2016).

Interestingly, we previously found that when co-applied with a psychological stressor, paraquat did not further augment corticosterone or neurochemical activity (Rudyk et al., 2015). This suggests that the toxicant and stressor do not synergistically or even additively reinforce each other’s actions, and while they may affect similar biological outcomes, they may do so through different mechanisms. Indeed, the oxidative stress factors, such as nitric oxide and superoxide, typically provoked by paraquat have been associated with HPA activation (Lee et al., 2013), but such factors are generally not induced by most stressors unless they are extremely severe and prolonged (e.g., 7 h or restraint or repeated restraint for weeks, compared to a typical 20–60 min acute session; García-Fernández et al., 2012; Lee et al., 2012; Moretti et al., 2013). Thus, we hypothesize that paraquat likely provokes HPA activation through its oxidative stress effects within the brain, whereas more psychologically relevant stressors signal through more classically prescribed stress pathways (e.g., by affecting limbic neurotransmission).

Overall, the present study indicates that paraquat and age can influence stress hormone and hippocampal functioning, as well as induce a range of behavioral adaptations over long periods of time. Some of the unexpected effects of paraquat are clearly in line with the perspective that repeated low-dose oxidative stress factors could promote adaptive responses over time. Although BDNF and Nrf2 were not affected, future studies should focus on other potential endogenous factors that might underlie paraquat-induced adaptive responses, including time-dependent variations in heat shock proteins or other molecular chaperones that contribute to cellular homeostasis. Whatever the case, the present data could have important implications for mechanisms of action of chronic oxidative stressors and might also help inform environmental policies regarding toxicant levels of exposure and age-dependent health risks.

## Author Contributions

SH and CAR conceived the study and prepared the manuscript. CAR, WC and JM conducted behavioral assays and western blot analysis. DL, ZD, KF and NP helped with biological assays and statistcal analyses.

## Conflict of Interest Statement

The authors declare that the research was conducted in the absence of any commercial or financial relationships that could be construed as a potential conflict of interest.
